# Different Behavioral Measures of Conditioned Magazine Activity Can Tell Different Stories about Brain Function

**DOI:** 10.1523/ENEURO.0560-24.2026

**Published:** 2026-04-21

**Authors:** Stephen G. Volz, Gabriel Loewinger, Inmaculada Marquez, Salvatore Fevola, Mihwa Kang, Ingrid Reverte, Anjali Krishnan, Matthew P. H. Gardner, Mihaela D. Iordanova, Guillem R. Esber

**Affiliations:** ^1^Department of Psychology, Brooklyn College, City University of New York, Brooklyn, New York 11210; ^2^Machine Learning Team, National Institute of Mental Health, NIH, Bethesda, Maryland 20892; ^3^Department of Psychology, La Ciénega University Center, University of Guadalajara, Ocotlán, Jalisco 47810, Mexico; ^4^ The Graduate Center, City University of New York, New York, New York 10016; ^5^ Department of Physiology and Pharmacology, Sapienza University of Rome, Rome 00185, Italy; ^6^Department of Psychology, CSBN/GRNC, Concordia University, Montreal, Quebec H4B 1R6, Canada

**Keywords:** conditioned magazine approach, cue–reward learning, neural bases of reward learning, pavlovian appetitive conditioning, principal component analysis, replicability

## Abstract

Elucidating the neural substrates of pavlovian reward learning requires reliable behavioral readouts. In conditioned magazine approach studies, rodents express reward expectancy by approaching the food magazine during cues that predict reward. This behavior is typically quantified using one of three measures: number of head entries, percentage of time in the magazine, or latency to respond. Yet these measures often diverge within the same discrimination task, making reliance on a single metric problematic. At the individual level, some animals express discrimination learning most clearly in one measure while showing little or no learning in the others, and animals may even switch their preferred measure across training. Reporting only one measure therefore risks underestimating the ability of a subset of animals. At the group level, sampling error can produce apparent differences across replications of the same design, limiting replicability. Moreover, brain manipulations can alter response topography, such that choosing one measure over another may lead to conflicting interpretations of neural function. To address this issue, we recommend reporting all raw behavioral measures and supplementing them with a dimensionality-reduction approach such as principal component analysis. Across multiple discrimination tasks in rats from both sexes, we show that subject-specific first principal component scores provide a composite index that more consistently reflects discrimination learning than any single raw measure. This approach enhances statistical power, improves reproducibility, and helps distinguish true learning deficits from changes in response topography. However, its broader application will require continued validation and careful consideration of its inherent methodological tradeoffs.

## Significance Statement

Accurately characterizing pavlovian reward learning requires reliable measurement of individual behavioral responses. In conditioned magazine approach studies, behavior is typically quantified by a single measure—such as head entries, time at the magazine, or response latency—but these measures often diverge. Reliance on one metric can underestimate discrimination ability, compromise reproducibility, and distort interpretations of neural manipulations. We show that applying principal component analysis to integrate multiple response measures yields a robust discrimination index that better reflects individual performance. This approach increases effect sizes, strengthens replicability, and reduces misinterpretation, providing scientific, economic, and ethical benefits for research on cue–reward learning.

## Introduction

Understanding how the brain controls behavior depends on reliable behavioral readouts. Yet even within the same procedure, different quantitative measures of a single cognitive variable can diverge within a subject across training, or disagree with one another across groups, leaving a precarious foundation for neuroscience inquiry. Such inconsistencies are evident in the conditioned magazine approach procedure (Boakes, 1977), the principal method for investigating the neural substrates of pavlovian reward learning in rodents (Ostlund and Balleine, 2007; Kusumoto-Yoshida et al., 2015; Lucantonio et al., 2015; Sharpe et al., 2017; Reverte et al., 2020).

**Figure 1.**
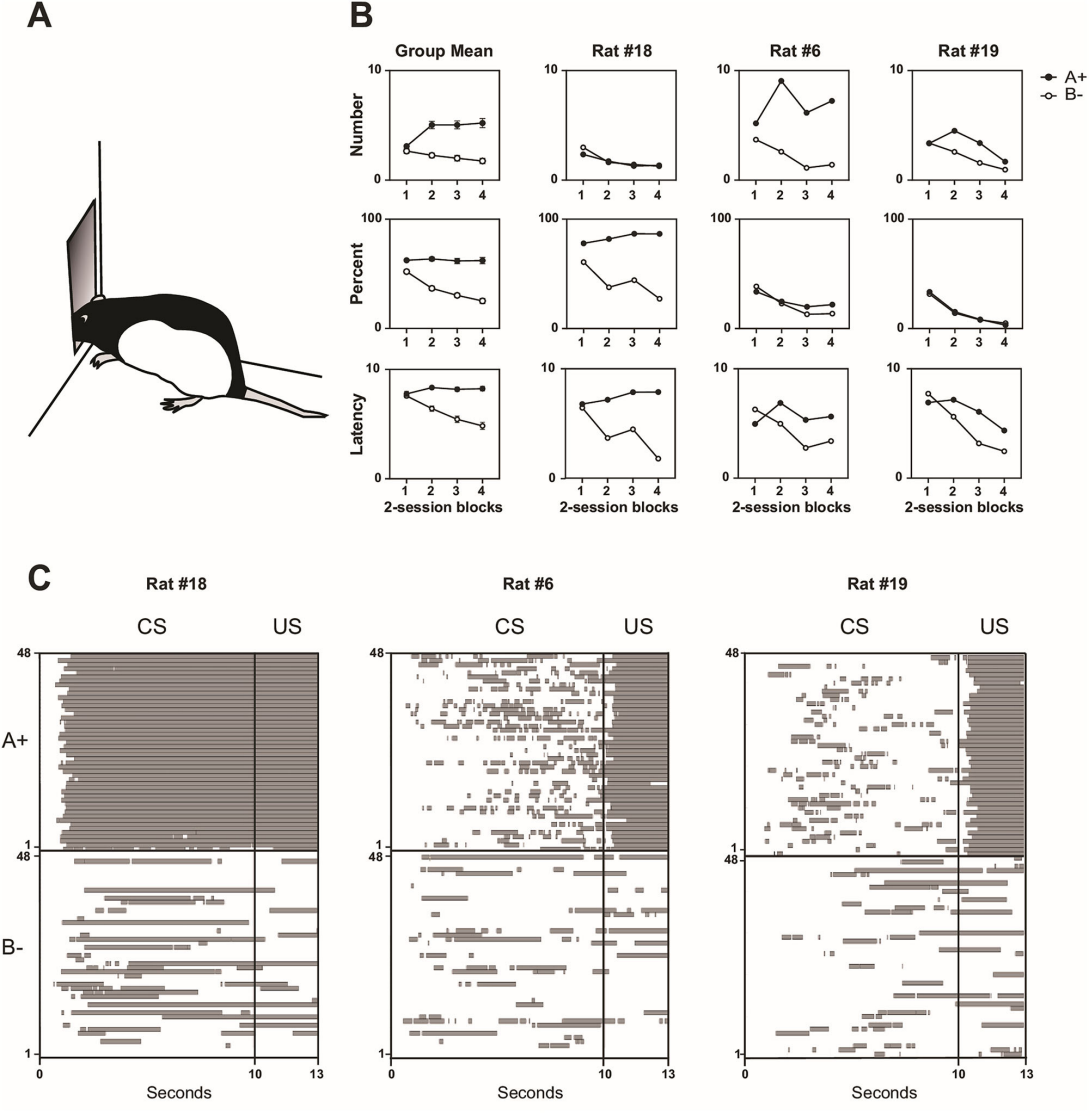
Individual differences in the behavioral expression of discrimination learning in the conditioned magazine approach procedure. ***A***, Magazine approach is commonly quantified in three ways: the number (or rate) of head entries during the cue, the percentage of cue time spent in the magazine, or the latency to enter the magazine after cue onset. ***B***, Discrimination performance in a visual discrimination task (A+ vs B−), expressed as head entries (top row), percent time (middle row), and latency (reverse-coded as cue duration – latency; bottom row). The symbols “+” and “−” indicate, respectively, the presence and absence of a sucrose reward. The left-column panels show group-averaged performance across rats (*n* = 32). Within-subject error bars were calculated with Cousineau (2005)’s method using Morey (2008)’s correction (O’Brien and Cousineau, 2014). The remaining panels show three example rats, each of which expressed discrimination learning clearly in only one measure, but weakly or not at all in the others. ***C***, Individual differences in response topography are further highlighted by patterns of magazine occupancy during the cue period across all A+ and B− trials in the final training session (trials plotted from bottom to top). Gray bars indicate periods in which the rat was in the magazine.

In this task, rodents are placed in conditioning chambers and presented with pavlovian cues that may signal delivery of an appetitive outcome (e.g., food pellets and sucrose solution) at a recessed magazine (Fig. 1*A*). A sensor at the entrance of the magazine records approach responses during cue presentation, reflecting the animal's reward expectancy. These responses are commonly quantified in one of three ways: the number (or rate) of head entries (Delamater, 1996; Kusumoto-Yoshida et al., 2015), the percentage of cue time spent in the magazine (Haselgrove et al., 2010; Esber and Holland, 2014), or the latency to approach after cue onset (Chaudhri et al., 2008; Lay et al., 2019). Each measure is assumed to index the same latent psychological construct—the degree of reward expectancy generated by the cue (i.e., associative strength or predictive value). As a result, it is common practice to report only one of them.

This practice is problematic. The three measures often tell different stories, even within the same subject. Prior work has noted discrepancies across experiments and attributed them to design differences such as reward rate. For instance, high reward rates favor behaviors best captured by percent-based metrics, whereas low reward rates favor count-based measures (Holland and Gallagher, 1993; Holland and Kenmuir, 2005; Burke et al., 2008; McDannald et al., 2011; Sharpe and Killcross, 2014; Sharpe et al., 2017). Here, we show that even under identical conditions, animals vary widely in how discrimination learning (i.e., distinguishing reinforced from nonreinforced cues) is expressed across measures (Experiment 1) and can even switch midtraining from one preferred measure to another (Experiment 2). Reporting only a single measure therefore risks underestimating the discrimination ability of a subset of animals. This increases statistical error, inflates sample sizes, and contravenes the principle of reduction in animal research. It also hampers replicability, since sampling error can yield groups biased toward different measures (Experiment 3), contributing to broader reproducibility challenges in neuroscience (Hensel, 2020). Furthermore, it can provide inaccurate behavioral correlates of neural activity and misrepresent the effects of neural manipulations (Experiment 4).

When multiple quantitative dependent variables are measured on observations nested within groups, a common approach in the behavioral sciences is multivariate analysis of variance (MANOVA). Compared with running separate ANOVAs, MANOVA can offer greater power and can detect associations among dependent variables. Like multiple regression and discriminant analysis, however, MANOVA is sensitive to multicollinearity, and therefore its use is limited in situations where the number of variables approaches or exceeds the number of observations. In repeated-measures studies with many within-subject conditions—such as factorial combinations of cues and sessions, as is typical in conditioned magazine approach studies—wide-format datasets create a separate column for each condition, rapidly inflating the number of variables relative to subjects. This inflation increases the risk of multicollinearity, which precludes the analysis, or of near multicollinearity, which provides unstable parameter estimates.

To further complicate matters, MANOVA requires nonzero variance within each group for every dependent variable. If all animals respond similarly to a particular cue on a given measure (e.g., by not responding to a nonreinforced cue), this measure will have zero variance and effectively become a constant, causing the MANOVA to fail. Even when this is not the case, conditioned magazine approach data often violate additional MANOVA assumptions: (1) The behavioral measures are typically correlated—a pattern leading to multicollinearity; (2) the measures rarely follow a multivariate normal distribution because they include bounded (e.g., percent time) or skewed variables; and (3) the assumption of homogeneous covariance matrices across groups is unlikely to hold when neural manipulations change the variability and interdependence of measures. Together, these issues make MANOVA impractical for many conditioned magazine approach designs—which may explain why it is rarely used in the literature (for an exception, see McDannald et al., 2011).

One method recently proposed as an alternative to MANOVA is to conduct a principal component analysis (PCA; Pearson, 1901; Jolliffe and Cadima, 2016) on the dependent variables and analyze the first principal component (PC1) with ANOVA (Abapihi et al., 2021). Here, we evaluate the merits of this approach in the conditioned magazine procedure. We show that when PCA is applied on a subject-specific basis to number of entries, percent time, and latency, it yields a composite index (PC1) that consistently captures each animal's ostensible discrimination ability. Importantly, our recommendation is not to replace direct measures with PC1, but to report all standard measures of magazine approach alongside subject-specific PC1 scores as a summary index of discrimination learning. We provide evidence that this approach can reveal effects obscured by reliance on a single measure, enhance replicability across samples, and more accurately characterize the impact of neural manipulations. By improving measurement sensitivity while preserving transparency, this method offers a rigorous and efficient tool for researchers using the conditioned magazine approach procedure. However, further validation across diverse discrimination settings is warranted. Moreover, because PC1 is a composite measure, its use entails some loss of direct interpretability and requires careful consideration of scaling and construct comparability issues.

## Materials and Methods

The studies presented here employ several variants of the conditioned magazine approach procedure. These variants differ in the type of reward (sucrose solution or food pellets) and in whether trials are delivered in the standard pavlovian manner or are self-initiated by the animal. In the self-initiated version, rats are required to turn on one of several possible cues chosen from a pseudorandomized list by performing a designated response, following the procedure described in Reverte et al. (2020). We previously demonstrated that cues trained under self-initiation transfer seamlessly to the standard procedure and thus function as pavlovian conditioned stimuli (Reverte et al., 2020). This procedural diversity provides a means to evaluate the consistency of standard magazine activity measures and the robustness of PC1 as a summary discrimination index across different task requirements. Below, we first describe general procedures common to all four studies, followed by experiment-specific details.

### Subjects

All studies employed gender-balanced, experimentally naive adult Long–Evans rats. The animals were bred from commercially available populations (Charles River Laboratories). They were housed individually in standard clear-plastic tubs (10.5 inch × 19 inch × 8 inch, Charles River Laboratories) with woodchip bedding. The colony room was maintained on a 14:10 light/dark cycle schedule. Behavioral sessions were conducted between 4 and 10 h after the onset of the light phase of the cycle. Throughout training, water access was restricted to 1 h/d following each experimental session while food was provided *ad libitum*. All animal care and experimental procedures were carried out in compliance with the ARRIVE guidelines (Percie du Sert et al., 2020) and the National Institutes of Health's Guide for the Care and Use of Laboratory Animals (Albus, 2012) and approved by the institutional Animal Care and Use Committee (Protocol #303). Further experiment-specific details are provided below.

### Apparatus

Behavioral training was conducted in eight modular conditioning chambers (32 cm long × 25 cm wide × 33 cm tall, Med Associates). Each chamber was enclosed in a ventilated sound-attenuating cubicle (74 cm × 45 cm × 60 cm) fitted with an exhaust fan that provided a background noise level of ∼50 dB. All reported locations of stimulus and response apparatus were measured from the grid floor of the conditioning chamber to the lowest point or edge of the apparatus. The left wall of the chamber housed a recessed food magazine (aperture: 5.1 cm × 15.2 cm) located at the bottom of the center panel 1.6 cm above the grid floor. This magazine was equipped with an infrared sensor for detecting head entries and connected to a pellet dispenser that could deliver 45 mg grain pellets. Flanking the food magazine, on the left and right panels, were two retractable levers located 6.5 cm from the grid floor, above each of which was a white jewel lamp (28 V DC, 100 mA) mounted 9.3 cm from the grid floor. Above each of these lamps was a speaker located 20.6 cm above the grid floor and connected to a dedicated tone generator capable of delivering a 2.5 Hz, 80 dB clicker (left panel) and a 70 dB white noise (right panel).

Two additional speakers were located on the left and right panels of the right wall of the chamber 24.8 cm above the grid floor. Each of them was also connected to a dedicated speaker capable of delivering a 12 kHz, 70 dB tone (left panel) and a 1 kHz, 80 dB tone (right panel). The right wall also housed a third jewel lamp located on the center panel 17.2 cm above the grid floor. Below this lamp, 4.6 cm above the grid floor, was a circular noseport 2.6cm in diameter, equipped with a yellow LED light and an infrared sensor for detecting nose entries. This noseport was flanked by a recessed liquid reward magazine (aperture: 5.1 cm × 15.2 cm) located on the right panel, 1.6 cm above the grid floor. The magazine was equipped with an infrared sensor for detecting head entries and connected to a liquid dipper that could deliver a 0.04 cc droplet of a 10% sucrose solution. The chambers remained dark throughout the experimental session except during presentations of the visual stimuli. In the same room was “a computer running Med-PC IV software” (Med Associates) on Windows OS which controlled and automatically recorded all experimental events via a fader control interface.

#### Magazine training

In all experiments, rats initially received one session of magazine training. In experiments employing a sucrose reward, rats with restricted water access (1 h daily) were trained to retrieve sucrose solution from the dipper cup. Each session began with a 2 min acclimation period in the conditioning chambers and lasted 62 min in total. Across 60 trials, the duration and frequency of sucrose availability were gradually reduced: During the first 10 trials, sucrose was available for 30 s every 30 s; during the next 20 trials, it was available for 20 s every 40 s; and during the final 30 trials, it was available for 10 s every 50 s. In the study involving food pellets as the reward (Experiment 2), rats were gradually food-deprived to 85% of their free-feeding weights prior to magazine training. A single magazine training session began with a 1 min acclimation period, after which one pellet was delivered into the food receptacle every minute for 30 min. The rats were left in the conditioning chamber for an additional 30 min with no scheduled events to allow them sufficient time to consume the pellets.

#### Shaping

In studies employing the self-initiated variant of the conditioned magazine approach procedure, rats underwent five shaping sessions during which they learned to perform the trial-initiation response during a trial-availability cue. In experiments using sucrose as the reward, this response consisted of a nose poke into a noseport adjacent to the sucrose magazine following illumination of the port light. During the first session, the noseport light was illuminated for up to 20 s. A nose poke during this period immediately terminated the light and led to 10 s of sucrose availability. Trials were separated by a variable intertrial interval (ITI; mean = 10 s; range, 5–15 s). If the rat failed to respond within 20 s, the light turned off, and the trial was repeated after a regular ITI. Across the next four shaping sessions, a response at the nose port triggered a progressively longer delay before sucrose delivery (2, 4, 6, and 8 s). During this delay, the noseport light flashed at 1 Hz (0.5 s on, 0.5 s off). In parallel, the duration of reward availability was gradually shortened to 8, 6, 4, and finally 3 s.

In experiments using food pellets as the reward, shaping followed the same general procedure, except that the trial-initiation response consisted of pressing a lever located to the right of the food magazine following lever insertion.

#### Discrimination tasks

Across studies, rats were trained on discrimination tasks that varied in their specific contingencies. These are described in detail in the methods for each experiment.

#### Dependent measures

Magazine activity was quantified in all studies as (1) the number of head entries during the cue, (2) the percent of cue time spent in the magazine, and (3) the latency to respond after cue onset. To align directionality across measures, latency was reverse-coded (cue duration – latency), such that higher scores indicated shorter latencies.

#### Principal component analyses

In each study, trial-by-trial data for number of entries, percent time, and latency were *z*-scored separately for each rat to place all measures on a common scale with a mean of zero. An alternative approach is to mean-center and normalize data by unit length. Because both methods differ only by a scaling factor, we adopted the more familiar *z*-score normalization to facilitate adoption of our approach. The accompanying analysis script allows users to choose between *z*-score and unit-length normalization (https://github.com/esberlab/Volz_et_al_PCA). A separate PCA was then conducted for each rat using MATLAB®'s *pca* function, and PC1 scores were selected for further statistical analyses. In Experiment 4, each PCA was conducted on the latter half of training (18 sessions) to avoid undue influence of early-session variability (e.g., intercue or intersession variance unrelated to discrimination performance, which emerged only in later sessions).

#### Statistical analyses

To compare the merits of PC1 scores with the standard behavioral measures, separate repeated-measures ANOVAs were conducted in *jamovi* with PC1 scores, number of entries, percent time, or reverse-coded latency scores as dependent variables (R Core Team, 2019; The jamovi Project, 2022). Significant interactions were followed up with post hoc pairwise comparisons using Tukey's HSD (Singmann et al., 2018; Lenth, 2020) or, where appropriate, with Bonferroni-corrected simple effects. All *jamovi* statistical tables are available at https://github.com/esberlab/Volz_et_al_PCA.

Session block-by-session block effect sizes (Hedges' *g*) for discriminative performance in terms of PC1 scores, number of entries, percent time, and latency were calculated using the *mes.m* script from the MES toolbox (Hentschke and Stüttgen, 2011), available at http://sourceforge.net/projects/mestoolbox/ and the MATLAB Central File Exchange (http://www.mathworks.com/matlabcentral/fileexchange/32398-measuresof-effect-size-toolbox). Where appropriate, within-subject error bars were calculated using the method of Cousineau (2005) with Morey’s (2008) correction (O’Brien and Cousineau, 2014).

#### Experiment 1: individual differences in the response topography of magazine approach

##### Subjects

Thirty-two experimentally naive, adult Long–Evans rats were used. They were run in four separate iterations in squads of eight rats each. The animals were between 20 and 34 weeks old by the start of the experiment, and their weights ranged between 257 and 329 g for females and 441 and 723 g for males.

##### Discrimination task

Experiment 1 employed the self-initiated variant of the conditioned magazine approach procedure, with sucrose as reward. Following magazine training and shaping, rats received eight sessions of simple discrimination training with two 10 s visual cues. Each session began with a 1 min acclimation period, after which trials became available. Trial availability was signaled by illumination of the noseport light for up to 20 s. A nose poke during this window terminated the light and immediately initiated one of the two visual cues. The cues consisted of (1) alternating flashes of the two jewel lamps on the left wall at 2 Hz (0.25 s on, 0.25 s off) and (2) steady illumination of the white jewel lamp on the right wall. Cue presentation followed a pseudorandom sequence with the constraint that no trial type occurred more than three times consecutively. One cue was consistently reinforced (A+), while the other was never reinforced (B−). On A+ trials, cue offset coincided with delivery of a 0.04 cc sucrose bolus (delay conditioning), which remained available for 3 s. Cues were counterbalanced across animals, and each could be presented 48 times in a session. Trials were separated by a variable ITI (mean = 10 s; range, 5–15 s). If a rat failed to initiate a trial within 20 s, the noseport light was terminated, and a new ITI began. Because rats self-paced their sessions, voluntary pauses could lengthen the effective ITI. Sessions ended when all scheduled trials were completed or after a 90 min time limit.

#### Experiment 2: adopting PC1 scores as an index of magazine approach protects against changes in response topography across training

##### Subjects

Two experimentally naive Long–Evans rats were used, labeled Rats #4 and #15. Rat #4 was male (430 g at the start of procedures), and Rat #15 was female (265 g).

##### Discrimination task

In this case study, Rat #4 was trained in the self-initiated variant of the conditioned magazine approach procedure, whereas Rat #15 was trained in the standard pavlovian variant. Both rats received food pellets as reward. Following magazine training—and, for Rat #4, additional shaping training—rats underwent discrimination training involving cues from two sensory modalities: visual (A, B) and auditory (C, D). Cue A was followed by six pellets (A → 6p) unless presented in compound with C, in which case the compound was followed by three pellets (AC → 3p). Cue B was never reinforced (B → 0p) unless presented in compound with D, in which case the compound was followed by one pellet (BD → 1p). Rat #4 received up to 24 presentations of each trial type (A → 6p, B → 0p, AC → 3p, BD → 1p) per session, whereas Rat #15 received 15 presentations of each, separated by a variable ITI (mean = 1 min; range, 30–90 s). For the present purposes, only the A → 6p versus B → 0p discrimination is shown and analyzed, as our goal is to characterize changes in response dynamics across training and to evaluate PC1 scores as a potential solution.

#### Experiment 3: adopting PC1 scores as an index of magazine approach enhances replicability

##### Subjects

Two replications of the same discrimination task included eight Long–Evans rats (four males, four females), tested approximately 2 months apart. Rats in the first replication were ∼22 weeks old, with weights of 475–533 g for males and 292–325 g for females. Rats in the second replication were ∼26 weeks old, with weights of 544–583 g for males and 311–340 g for females.

##### Discrimination task

Experiment 3 employed the standard variant of the conditioned magazine approach procedure, with sucrose as reward. Following magazine training, rats received 40 sessions of training on a positive-patterning discrimination of the form AB → sucrose, A → nothing, and B → nothing, where A and B were visual and auditory cues, respectively. Each trial type was presented 16 times per session, with a mean ITI of ∼13 s. Training also included a negative-patterning discrimination (CD → nothing, C → sucrose, D → sucrose), with C and D likewise assigned to visual and auditory modalities. These results are excluded from the present report as they add nothing to the present concern. For the same reason, we do not include data from a pretraining phase conducted only in the first iteration, in which animals received C → sucrose and A → nothing trials. This phase accounts for the initially low responding to Cue A during positive-patterning training. The present replications formed part of a larger study reported previously (Kang et al., 2021).

#### Experiment 4: PC1 scores can help distinguish true discrimination deficits from changes in response topography induced by neural manipulations

##### Subjects

Twenty-four gender-balanced Long–Evans rats were employed, approximately 5 months old at the start of the study. Six animals (four males, two females) were excluded on histological grounds, leaving nine rats in the lesion group and nine in the sham group. Prior to surgery, body weights ranged from 283 to 624 g in the lesioned animals and 262 to 619 g in the shams.

### Surgery

Surgical procedures were conducted under isoflurane anesthesia and aseptic conditions. Rats assigned to the lesion group received bilateral ventral striatal lesions targeting the nucleus accumbens core and shell. Coordinates relative to bregma were AP +2.1 mm, ML ±1.6 mm, and DV −7.2 mm (from skull surface). Two small holes were drilled above the target sites, and *N*-methyl-d-aspartic acid (NMDA; Sigma-Aldrich) dissolved in saline (20 µg/µl) was infused over 3 min using a 2.0 µl Hamilton syringe at a rate of 0.1 µl/min. Following infusion, the needle was left in place for 10 min to permit diffusion before being withdrawn, and the holes were sealed with bone wax (Surgical Specialties). Sham animals underwent the same surgical procedures, except that the needle was lowered to the target coordinates for the same duration without infusion.

#### Discrimination task

Experiment 4 employed the self-initiated variant of the conditioned magazine approach procedure, with sucrose as reward. Following a 2-week postoperative recovery period, rats were water restricted, magazine trained, and shaped to self-initiate trials. They then underwent positive-patterning discrimination training across 36 sessions, following the procedure outlined for Experiment 3. Only data from the latter 18 sessions are presented, as there was little evidence of discriminative performance before then.

#### Histology

After behavioral testing, rats were killed with a lethal dose of Euthanasia-III (Med-Pharmex) and transcardially perfused with 0.9% saline followed by 10% buffered formalin. Brains were extracted, postfixed in 10% buffered formalin for 24 h, and cryoprotected in 30% sucrose PBS. Coronal sections (40 µm) were sliced, mounted, and stained with cresyl violet. Sections were imaged and matched to a series of coronal plates (+3.00, +2.56, +2.16, +2.04, +1.56, +0.96, +0.60 mm from bregma) encompassing the target region, based on the Paxinos and Watson (2007) atlas. Lesions were characterized in Photoshop by the absence of visible neuronal bodies, the presence of gliosis, and evidence of tissue collapse (e.g., shrinkage or ventricular expansion due to proximity to the lateral ventricles). The extent of target damage was quantified in ImageJ as the percentage of overlap between the demarcated lesion and the anatomical boundaries of the target region on each section (Fig. 7*A*). Animals were considered to have acceptable lesions if they exhibited ≥50% damage on average across the selected coronal plates.

## Results

### Experiment 1: individual differences in the response topography of magazine approach

The left panels of Figure 1*B* show the performance of a group of rats faced with a simple visual discrimination (Light A → sucrose; Light B → nothing). At the group level, all three measures appear to align: On average, rats make more entries (top), spend more time in the magazine (middle), and respond more quickly (bottom; shown as reverse-coded latency: cue duration – latency) during the reinforced cue than the nonreinforced cue. This apparent agreement at the group level, however, conceals striking individual differences in response topography, as illustrated in the right panels of Figure 1*B*. The examples show the performance of three individual rats across the three measures. Rat #18 clearly discriminates when assessed by percent time or latency, but not by number of entries. Rat #6 shows the opposite pattern, solving the discrimination when measured by entries and latency, but less convincingly by percent time. Rat #19 discriminates best in latency, less so in entries, and not at all in percent time.

**Figure 2.**
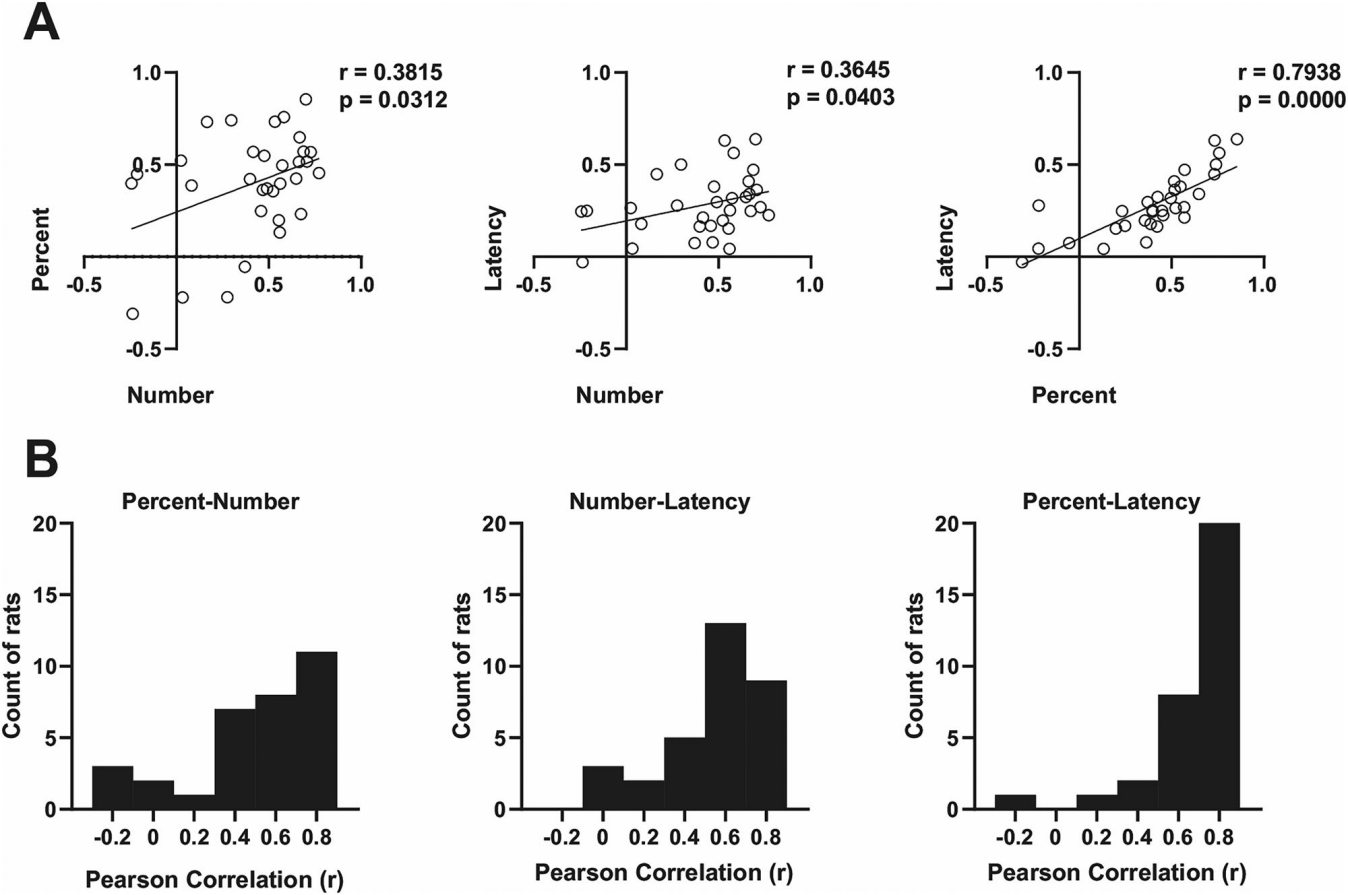
***A***, Correlations between standard behavioral measures of magazine approach on the last session block of A+ B− discrimination training. For each measure, a discrimination index was computed for each of the 32 rats from trial-averaged data using the formula (A − B)/(A + B), where ***A*** and ***B*** represent response scores to cues A+ and B−. Positive values indicate stronger responding to A+ (successful discrimination), negative values indicate stronger responding to B−, and values near zero indicate indifference. Pearson’s correlations were then calculated across animals for each pair of behavioral measures. Despite overall positive associations, the scatterplots reveal substantial individual differences in response topography. ***B***, Distribution of within-subject correlations between discrimination indices derived from each pair of behavioral measures. Notably, the two most widely used measures in the literature—number of entries and percent time—produced the largest number of cases with weak or even negative correlations.

Figure 1*C* depicts the moment-to-moment magazine activity of these rats across all trials of a representative session, providing further insight into their response topography. Rat #18 consistently enters the magazine soon after Cue A onset and remains there until reward delivery. In contrast, Rat #6 makes shorter, sporadic visits, and Rat #19 tends to enter the magazine earlier during reinforced trials. Importantly, such divergent response profiles are not rare exceptions. To demonstrate this, we calculated discrimination indices of the form (A − B)/(A + B) based on trial-averaged performance during the final two-session block for each behavioral measure. For each rat, average responding to reinforced (A → sucrose) and nonreinforced (B → nothing) cues was first computed separately for each measure, and these averages were then used to derive the discrimination index. We plotted the indices for each rat across all pairwise combinations of measures (Fig. 2). Positive values along either axis indicate stronger responding to the reinforced cue, negative values indicate stronger responding to the nonreinforced cue, and values near zero reflect little or no discrimination. At the group level (Fig. 2*A*), the two most commonly reported measures—number of entries and percent time—were only moderately correlated (*r* = 0.38, *p* = 0.031). This pattern likely reflects differences in response style: Rats that insert their heads deeply into the magazine tend to accumulate high percent time scores but few entries, whereas rats that hover at the magazine threshold produce more entries but less sustained occupancy. A similarly moderate correlation was observed between number of entries and latency (*r* = 0.36, *p* = 0.040), whereas percent time and latency showed a much stronger association (*r* = 0.79, *p* = 0.0001). At the individual level (Fig. 2*B*), although many rats displayed positive correlations across measures, others showed weak or even negative correlations, underscoring the heterogeneity of response topographies.

These findings raise the question of how to best capture each animal's discrimination ability while acknowledging individual differences in response topography. Our starting point was the observation that the behavioral measure best capturing discrimination within a given subject also tended to show the largest marginal variance. Once a discrimination emerges, much of the variance in that measure likely reflects differences in responding to reinforced versus nonreinforced cues. We reasoned that the information across all three measures could be leveraged by creating a linear combination that maximizes explained variance, thereby producing a composite index of discrimination learning for each subject.

Principal component analysis (PCA) provides such a solution. PCA is a multivariate technique that transforms potentially correlated variables into orthogonal components, each accounting for a proportion of the total variance in the data (Pearson, 1901; Jolliffe and Cadima, 2016). The first principal component (PC1) captures the largest share of variance and can be interpreted based on its relationship to the original measures. Importantly, the rationale for this approach—namely, that the primary source of variance arises from intercue differences—applies specifically to discrimination learning, and not to cases where cues receive identical reinforcement (e.g., Light A → sucrose; Light B → sucrose).

**Figure 3.**
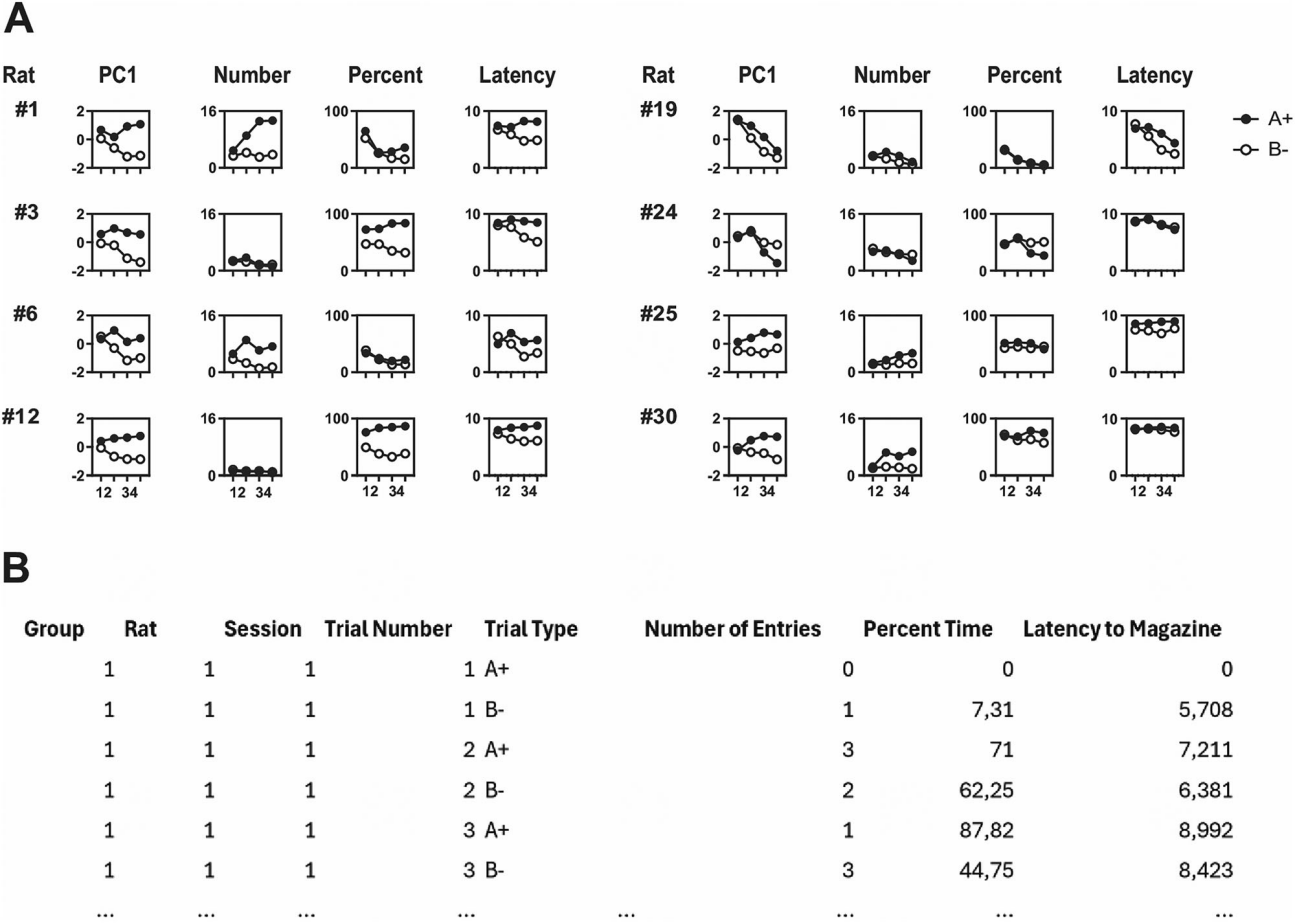
***A***, Initial rows of the input matrix we used to calculate rat-specific PCAs, containing each animal's scores in terms of number of entries, percent time, and latency to respond, organized by session, trial, and cue type. ***B***, Performance in the A+ versus B− discrimination task in a subset of rats, plotted in terms of PC1 scores, number of entries, percent time, and latency to respond. These examples illustrate how PC1 scores tended to align with the behavioral measure that best captured each rat's discrimination ability, highlighting their value as a composite index of discrimination learning.

To evaluate PC1 scores as a potential index of discrimination, we performed a subject-specific PCA for each of the 32 rats trained on the simple visual discrimination shown in Figure 1*B*. The input matrix for each PCA consisted of the animal's trial-by-trial scores across all three measures, organized by session, trial number, and trial type. A representative portion of this matrix is shown in Figure 3*A*.

#### Individual-level analyses

A critical test of PC1 scores as an index of magazine activity is how well they capture discrimination learning at the individual level. Figure 3*A* illustrates discrimination performance for a subset of rats in terms of PC1 scores, number of entries, percent time, and latency (data for all 32 rats are available at https://github.com/esberlab/Volz_et_al_PCA). Consistent with our earlier observations, rats varied in their response topography; that is, in how they expressed discrimination learning across the three raw measures. Importantly, PC1 scores generally aligned with the measure that provided the clearest cue discrimination for a given subject. As expected, intersession variability in responding also contributed to these scores (e.g., Rats #1, #19, and #30).

Critically, several rats that failed to show discrimination according to one or more raw measures—such as number of entries (Rats #3, #12), percent time (Rats #6, #19, #25), or latency (Rat #30)—nonetheless exhibited clear discrimination when assessed by PC1 scores. If only a single measure had been considered, these animals might have been misclassified as nonlearners and likely dismissed as outliers. Conversely, rats that performed poorly according to PC1 scores also showed no evidence of discrimination across any of the raw measures (e.g., Rat #24), justifying their classification as true outliers. This suggests that PC1 scores minimize “false negatives” (animals wrongly judged not to discriminate), providing a more faithful subject-specific index of discrimination learning.

**Figure 4.**
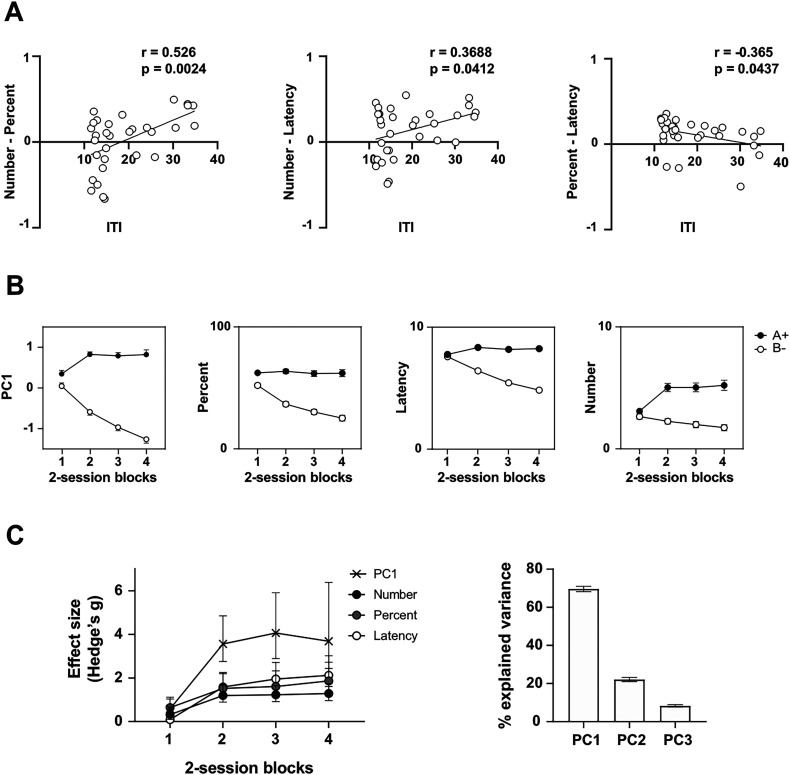
***A***, Relationship between response topography and reward rate (ITI). For each rat and each pair of behavioral measures, we computed the difference between discrimination indices to generate an index of whether learning was expressed more strongly in one measure versus the other. These difference scores were then plotted against the animal's average intertrial interval (ITI) during the final session block (a measure inversely related to reward rate). ***B***, PC1 scores optimize detection of discrimination learning at the group level. Mean responding during the A+ versus B− discrimination is shown for PC1 scores, number of entries, percent time, and latency to respond. Within-subject error bars were calculated using Cousineau (2005)’s method with Morey (2008)’s correction. ***C***, Left panel, Effect sizes of discrimination across training for PC1 scores compared with the direct behavioral measures. Right panel, Proportion of variance explained by each principal component.

As noted above, one factor that influences the response topography of magazine approach is the frequency of reward encounters within a session. Prior work has shown that higher reward rates encourage percent-based responding, whereas lower rates promote number-based performance (Holland and Gallagher, 1993; Holland and Kenmuir, 2005; Burke et al., 2008; McDannald et al., 2011; Sharpe and Killcross, 2014; Sharpe et al., 2017). In our study, rats self-paced their trials, raising the possibility that animals working at a faster pace (and thus receiving rewards more frequently) might express discrimination primarily in terms of percent time, whereas slower-working animals might favor number of entries. To test this, we computed the difference between each rat's number-based and percent-based discrimination indices [(A − B)/(A + B)] during the final session block. Positive values indicate stronger discrimination based on entries, while negative values indicate stronger discrimination based on percent time. We then plotted this difference against the mean ITI for the same session block, which is inversely related to reward rate (i.e., longer ITIs imply lower reward rates; Fig. 4*A*, left panel). Consistent with prior findings, we observed a significant positive correlation (*r* = 0.526, *p* = 0.0024), demonstrating that discrimination learning was more likely to be expressed in number of entries at lower reward rates and in percent time at higher reward rates. However, the figure also reveals that not all rats experiencing high reward rates showed the discrimination more clearly in percent time. Thus, reward rate cannot fully account for individual differences. This interpretation is reinforced by the observation that identical reward rates can yield different response topographies across groups (Experiment 3) and even across sessions within the same animal (Experiment 2).

For completeness, we also compared discrimination indices based on latency to respond with those derived from number of entries (Fig. 4*A*, middle panel) and percent time (Fig. 4*A*, right panel). Both analyses yielded significant correlations. As reward rate decreased, rats were more likely to express discrimination through latency rather than entries (*r* = 0.369, *p* = 0.041; Fig. 4*A*, middle panel). Conversely, at high reward rates, discrimination was expressed more clearly in latency than in percent time, although this difference diminished as reward rate declined (*r* = 0.365, *p* = 0.044; Fig. 4*A*, right panel). These results are noteworthy and, to our knowledge, novel, given that latency to respond is rarely reported as a dependent variable in magazine approach studies.

#### Group-level analyses

In behavioral neuroscience, differences between conditions are typically evaluated at the group level. Figure 4*B* shows group performance in terms of PC1 scores compared with the three direct behavioral measures. Data were averaged first across trials within two sessions and then across rats. As expected, PC1 accounted for the majority of variance relative to PC2 and PC3 (Fig. 4*C*, right), making it the most informative component for assessing discrimination learning. Although all three raw measures provided evidence of discrimination learning, PC1 scores consistently yielded the largest effect sizes (effect of cue: PC1, *F*_(1,31)_ = 223.67, *p* < 0.001, η^2^p = 0.88; number, *F*_(1,31)_ = 33.95, *p* < 0.001, η^2^p = 0.52; percent, *F*_(1,31)_ = 76.4, *p* < 0.001, η^2^p = 0.71; latency, *F*_(1,31)_ = 163.8, *p* < 0.001, η^2^p = 0.84. Cue × session block interaction: PC1, *F*_(3,93)_ = 79.46, *p* < 0.001, η^2^p = 0.72; number, *F*_(3,93)_ = 26.57, *p* < 0.001, η^2^p = 0.46; percent, *F*_(3,93)_ = 32.6, *p* < 0.001, η^2^p = 0.51; latency, *F*_(3,93)_ = 65.6, *p* < 0.001, η^2^p = 0.68).

Post hoc comparisons of A+ versus B− trials across session blocks confirmed that, except on the first session block, PC1 scores outperformed the raw measures. Results for each measure were as follows. PC1: Session block 1, *t*_(31)_ = 3.15, *p* = 0.063; Session block 2, *t*_(31)_ = 14.33, *p* < 0.001; Session block 3, *t*_(31)_ = 14.05, *p* < 0.001; Session block 4, *t*_(31)_ = 13.06, *p* < 0.001. Number: Session block 1, *t*_(31)_ = 2.64, *p* = 0.180; Session block 2, *t*_(31)_ = 5.69, *p* < 0.001; Session block 3, *t*_(31)_ = 5.65, *p* < 0.001; Session block 4, *t*_(31)_ = 5.85, *p* < 0.001. Percent: Session block 1, *t*_(31)_= 4.43, *p* = 0.002; Session block 2, *t*_(31)_ = 9.15, *p* < 0.001; Session block 3, *t*_(31)_ = 8.01, *p* < 0.001; Session block 4, *t*_(31)_ = 8.24, *p* < 0.001. Latency: Session block 1, *t*_(31)_ = 0.62, *p* = 0.998; Session block 2, *t*_(31)_ = 11.76, *p* < 0.001; Session block 3, *t*_(31)_ = 11.90, *p* < 0.001; Session block 4, *t*_(31)_ = 11.36, *p* < 0.001. Consistent with these results, Hedges’ *g* values showed that PC1 scores produced the largest effect sizes for every block except the first (Fig. 4*C*, left).

Some caution is warranted when comparing performance across measures. The outcome variables differ in distributional properties (e.g., latency is continuous, and entries are discrete), and PCA combines them through linear transformations without explicitly propagating uncertainty from the estimation of the principal components. Thus, effect-size comparisons between raw measures and PC1 should be interpreted with that caveat in mind. Nonetheless, the results underscore the potential of PC1 scores to provide greater sensitivity in detecting discrimination learning, especially when individual differences in response topography might otherwise obscure it. This increased sensitivity suggests that PC1 scores could enable researchers to reduce the length of discrimination training and achieve reliable results with smaller sample sizes.

### Experiment 2: adopting PC1 scores as an index of magazine approach protects against changes in response topography across training

**Figure 5.**
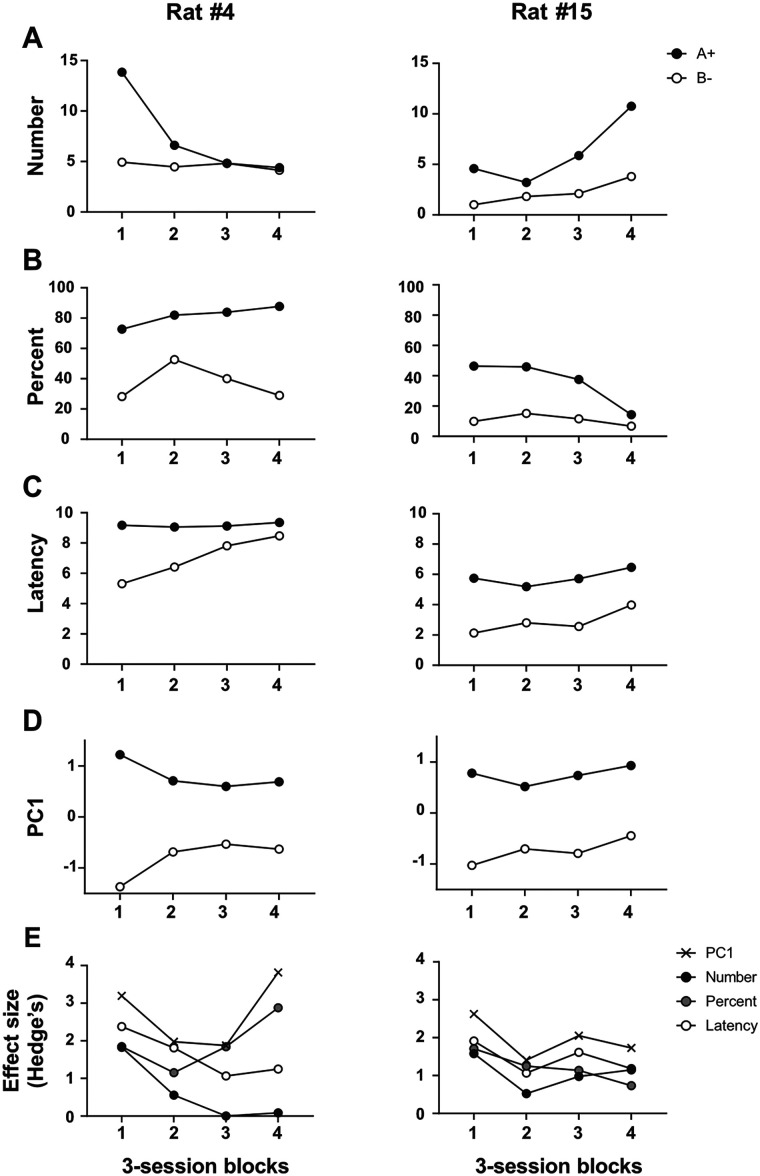
PC1 scores are relatively unaffected by midtraining changes in response topography. ***A–C***, Examples of shifting response patterns in two rats trained on an A+ versus B− visual discrimination embedded within a more complex task (see Materials and Methods, Experiment 3: adopting PC1 scores as an index of magazine approach enhances replicability). Here, the “+” symbol indicates delivery of a six-pellet reward. ***D***, Discrimination performance expressed as PC1 scores. ***E***, Session block-by-session block comparison of discrimination effect sizes derived from PC1 scores versus the three direct behavioral measures.

Although Figure 3*B* might suggest that performance within each behavioral measure remains stable once a discrimination is acquired, we have observed dynamic patterns of responding across training sessions. This is illustrated in Figure 5, which shows the performance of two rats trained on a simple visual discrimination (A → 6 pellets, B → nothing) over 12 sessions, as part of a larger discrimination task (details omitted here; see Materials and Methods). Rat #4 (Fig. 5, left column) initially displays robust discrimination across all three raw measures, but performance gradually deteriorates when assessed by number of entries or latency. In contrast, Rat #15 (Fig. 5, right column) shows a progressive deterioration in discrimination performance when evaluated by percent time. These shifts in response topography are obscured when only a single behavioral measure is reported, which can lead to misclassification of animals as outliers or dilution of group effects. In this study, we examine whether PC1 scores offer a more reliable characterization of discrimination ability under such circumstances.

The left and right panels of Figure 5*D* show discrimination performance for each rat based on PC1 scores. According to this index, both animals successfully discriminated in the first three-session block and, importantly, maintained this performance across the entire training period. Figure 5*E* presents session block-by-session block comparisons of effect sizes for PC1 scores versus the three raw behavioral measures. In both rats, PC1 consistently produced the largest effect sizes, indicating that it is relatively robust to midtraining shifts in response topography and provides a more stable measure of discrimination ability across training.

The variance explained by the components further supports the utility of PC1. For Rat #4, PC1 accounted for 61.3% of the variance, compared with 27.5% for PC2 and 11.1% for PC3. For Rat #15, PC1 explained 71.6% of the variance, with PC2 and PC3 accounting for 22.9 and 5.5%, respectively.

### Experiment 3: adopting PC1 scores as an index of magazine approach enhances replicability

Individual differences in the response topography of magazine approach imply that sampling error can lead different groups of rats, even under identical training conditions, to express discrimination ability more strongly in one behavioral measure than another. This variability poses a replicability challenge both within and across laboratories.

**Figure 6.**
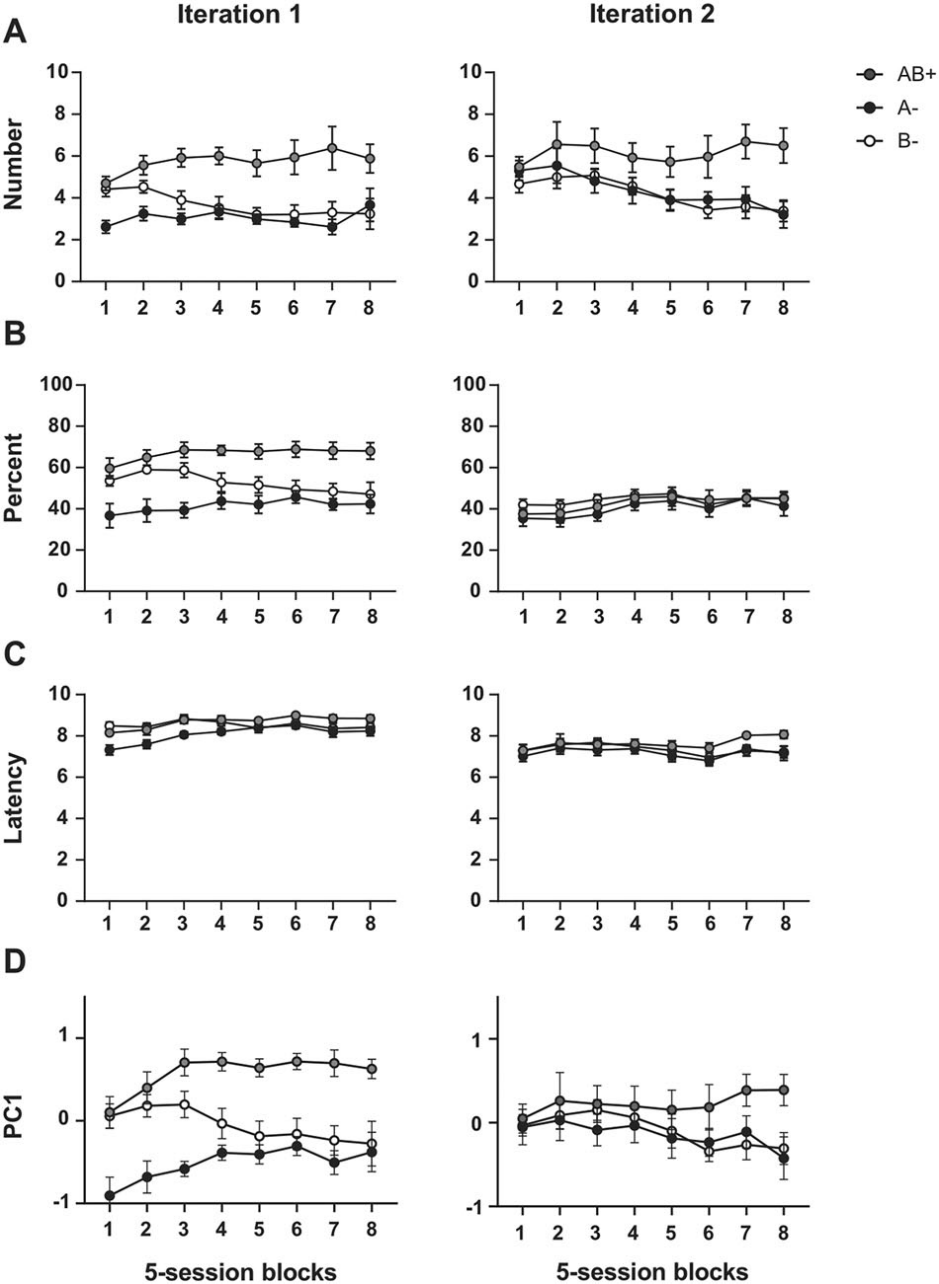
PC1 scores mitigate replicability issues that arise when only a single behavioral measure is reported. ***A–C***, Results of two independent iterations of a positive-patterning discrimination (AB + A− B−), in which Cues A and B (visual and auditory, respectively) were reinforced with sucrose only when presented in compound. Both iteration groups solved the discrimination when assessed by number of entries, and neither did so when assessed by latency to respond. However, only the first iteration group solved the task in terms of percent time. ***D***, PC1 scores captured the discrimination ability of both iteration groups, providing a more consistent index across replications.

An example comes from two iterations of the same positive-patterning discrimination conducted in our laboratory (AB → sucrose; A → nothing; B → nothing, with A as a light and B as a tone; Fig. 6). In the first iteration (Fig. 6*A*), rats clearly solved the discrimination when evaluated by number of entries and percent time (left panels). In the second iteration (Fig. 6*B*), however, the group failed to solve it when assessed by percent time (right panels). Latency measures provided no clear evidence of discrimination in either iteration. This pattern of results showcases how reliance on a single behavioral measure across replications is vulnerable to sampling error, as different groups may favor different topographies of responding. In this study, we test whether using PC1 scores as a common index across replications mitigates this risk and improves replicability.

Figure 6 shows the results of the positive-patterning discrimination across the two iterations, shown separately for each of the three behavioral measures and for PC1 scores. Next, we report the results of parallel statistical analyses conducted on each of them.

#### Number of entries

As noted above, both iteration groups successfully solved the discrimination when evaluated by number of entries (Fig. 6*A*). This was supported by a significant main effect of cue, *F*_(2,28)_ = 13.60, *p* < 0.001, η^2^p = 0.49, and a cue × session block interaction, *F*_(14,196)_ = 3.02, *p* < 0.001, η^2^p = 0.18. No other effects or interactions were significant, including those involving group, confirming that rats in both replications solved the discrimination on this measure. Simple effects analyses at the terminal stage of training (Session block 8) reinforced this conclusion. In the first iteration, rats entered the magazine marginally more during AB+ than A−, *t*_(7)_ = 2.10, *p* = 0.074, Cohen's *d* = 0.74, and B−, *t*_(7)_ = 2.13, *p* = 0.070, Cohen's *d* = 0.76. In the second iteration, rats likewise entered more during AB+ than A−, *t*_(7)_ = 2.51, *p* = 0.040, Cohen's *d* = 0.89, and B−, *t*_(7)_ = 2.54, *p* = 0.038, Cohen's *d* = 0.90.

#### Percent time

An equivalent analysis conducted on the percent time data (Fig. 6*B*) highlighted a potential replicability issue for this measure across iterations. The analysis revealed significant effects of cue (*F*_(2,28)_ = 10.97, *p* < 0.001, η^2^p = 0.44) and an iteration group × cue interaction (*F*_(2,28)_ = 7.58, *p* *=* 0.002, η^2^p = 0.35). Simple main effects separately conducted for each iteration on Session block 8 revealed that while rats in the first iteration spent more time in the magazine on AB+ than A− (*t*_(7)_ = 3.57, *p* = 0.01, Cohen's *d* = 1.26) and B− trials (*t*_(7)_ = 3.10, *p* = 0.018, Cohen's *d* = 1.10), rats in the second iteration failed to do so (AB+ vs A−, *t*_(7)_ = 0.60, *p* = 0.566, Cohen's *d* = 0.21; AB+ vs B−, *t*_(7)_ = −0.05, *p* = 1, Cohen's *d* = 0.02).

#### Latency

An equivalent analysis conducted on the latency data revealed that, across iterations, rats solved the discrimination according to this measure (Fig. 6*C*). There was a significant main effect of cue (*F*_(2,28)_ = 7.80, *p* = 0.002, η^2^p = 0.36) and a cue × session block interaction (*F*_(14,196)_ = 3.22, *p* < 0.001, η^2^p = 0.19). Simple main effects separately conducted for each iteration group on Session block 8 revealed that rats in the first iteration entered the magazine earlier after cue onset on AB+ than A− (*t*_(7)_ = 2.83, *p* = 0.026, Cohen's *d* = 1.00) or, marginally, B− trials (*t*_(7)_ = 2.03, *p* = 0.082, Cohen's *d* = 0.72). Rats in the second iteration also entered the magazine earlier on A+ than A− (*t*_(7)_ = 3.68, *p* = 0.008, Cohen's *d* = 1.30) or B− trials (*t*_(7)_ = 2.78, *p* = 0.028, Cohen's *d* = 0.98).

#### PC1

On average, PC1 accounted for 63.2% of the variance in the first iteration (compared with 24.5% for PC2 and 12.2% for PC3) and 61.1% in the second iteration (compared with 23.7% for PC2 and 15.3% for PC3). Analysis of PC1 scores suggested that this index captured discrimination performance more effectively than the direct behavioral measures (Fig. 6*D*). There was a significant main effect of cue, *F*_(2,28)_ = 14.46, *p* < 0.001, η^2^p = 0.51, as well as significant cue × group, *F*_(2,28)_ = 3.48, *p* = 0.045, η^2^p = 0.20; cue × session block, *F*_(14,196)_ = 4.34, *p* < 0.001, η^2^p = 0.24; and group × cue × session block interactions, *F*_(14,196)_ = 1.84, *p* = 0.035, η^2^p = 0.12. Simple effects analyses conducted for Session block 8 showed that rats in the first iteration responded more during AB+ than A− trials, *t*_(7)_ = 4.16, *p* = 0.004, Cohen's *d* = 1.47, and more during AB+ than B− trials, *t*_(7)_ = 2.96, *p* = 0.022, Cohen's *d* = 1.05. Rats in the second iteration showed a similar, although marginally significant pattern (AB+ vs A−, *t*_(7)_ = 2.32, *p* = 0.054, Cohen's *d* = 0.82; AB+ vs B−, *t*_(7)_ = 2.36, *p* = 0.050, Cohen's *d* = 0.84). These results demonstrate how PC1 scores can mitigate replicability challenges that arise from variability in how discrimination learning is expressed across iterations.

### Experiment 4: PC1 scores can help distinguish true discrimination deficits from changes in response topography induced by neural manipulations

**Figure 7.**
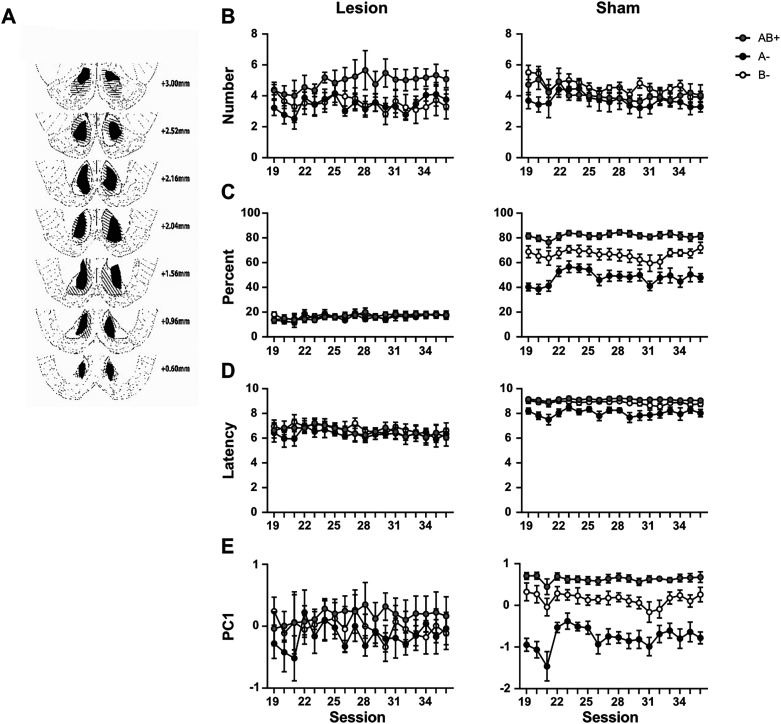
PC1 scores help resolve discrepancies in response topography induced by neural manipulations. ***A***, Histological reconstructions of ventral striatal lesions. The black and gray areas represent the estimated minimum and maximum lesion confines, respectively, at various coronal planes (Paxinos and Watson, 2007). ***B–D***, Discrimination performance in lesioned (left) and sham-operated (right) rats trained on a positive-patterning discrimination (AB + A− B−), where visual Cue A and auditory Cue B predicted sucrose only when presented in compound. Lesioned rats appeared to perform better than shams when assessed by number of entries, but worse when assessed by percent time, with latency scores aligning more closely with the percent time measure. Reliance on any single behavioral measure could therefore lead to opposite conclusions about the effects of the lesions. ***E***, PC1 scores provided a more accurate index of discrimination learning by combining information across measures, thereby accommodating lesion-induced shifts in response topography.

Brain manipulations can introduce additional variability in response topography, shifting the expression of discrimination learning from one behavioral measure to another relative to controls. If only a single measure is monitored, such shifts risk being misinterpreted as differences in discrimination ability rather than changes in response strategy. This concern is illustrated by a pilot study in which rats were trained on the same positive-patterning discrimination described in Experiment 3 after receiving ventral striatum or sham lesions (Fig. 7). Strikingly, the results suggest that selecting one behavioral measure of magazine activity over another can, in extreme cases, lead to opposite conclusions about neural function. The purpose of this study is to highlight this issue and underscore the importance of reporting multiple behavioral measures. In addition, we provide preliminary evidence that supplementing those raw measures with PC1 scores will help distinguish between manipulation-induced shifts in response topography and genuine deficits in discrimination learning.

Panels B–E of Figure 7 show average group performance across the last 18 training sessions, expressed as number of entries, percent time, latency to respond, and PC1 scores. We focused on the latter half of training to prevent PC1 scores from being unduly influenced by early variance unrelated to the late solution of the discrimination. The results of parallel statistical analyses conducted on each of these three raw measures and on PC1 scores are reported below.

#### Number of entries

Inspection of Figure 7*B* suggests that, while sham-operated rats did not reliably solve the discrimination when assessed by number of entries, rats with ventral striatum lesions appeared to perform somewhat better. This was reflected in a trend-level group × cue × session interaction (*F*_(34,544)_ = 1.37, *p* = 0.084, η^2^p = 0.08), with no other significant main effects or interactions. To assess performance at the end of training, simple effects were examined separately for each group using the mean of the final five sessions. Lesioned animals showed modest evidence of discrimination, entering the magazine more often on AB+ than A− trials (*t*_(8)_ = 2.50, *p* = 0.036, Cohen's *d* = 0.83), although not significantly more than on B− trials (*t*_(8)_ = 1.68, *p* = 0.132, Cohen's *d* = 0.56). In contrast, sham animals did not enter the magazine more often during AB+ compared with either A− (*t*_(8)_ = 0.67, *p* = 0.522, Cohen's *d* = 0.22) or B− (*t*_(8)_ = −0.65, *p* = 1, Cohen's *d* = 0.22).

#### Percent time

Strikingly, examination of percent time scores (Fig. 7*C*) revealed a different pattern of results. In this measure, ventral striatum lesions were associated with poorer discrimination performance, reflected in a significant group × cue interaction (*F*_(2,32)_ = 20.04, *p* < 0.001, η^2^p = 0.56). Significant main effects were also observed for group (*F*_(1,16)_ = 109, *p* < 0.001, η^2^p = 0.87), cue (*F*_(2,32)_ = 22.38, *p* < 0.001, η^2^p = 0.58), and session (*F*_(17,272)_ = 1.90, *p* = 0.018, η^2^p = 0.11), with no other significant interactions. Simple effects analyses at the terminal point of training (mean of the last five sessions) indicated that sham-operated rats spent more time in the magazine on AB+ trials compared with both A− (*t*_(8)_ = 7.93, *p* < 0.002, Cohen's *d* = 2.64) and B− trials (*t*_(8)_ = 3.21, *p* = 0.012, Cohen's *d* = 1.07). In contrast, lesioned rats did not discriminate between reinforced and nonreinforced trial types (AB+ vs A−, *t*_(8)_ = 0.24, *p* = 0.814, Cohen's *d* = 0.08; AB+ vs B−, *t*_(8)_ = 0.09, *p* = 0.932, Cohen's *d* = 0.03). The discrepancy between the number of entries and percent time measures illustrates how neural manipulations can alter the topography of magazine approach, underscoring the need to monitor and report multiple behavioral indices.

#### Latency

Inspection of Figure 7*D* suggests that sham rats may have discriminated better than lesioned rats in terms of latency scores, consistent with the percent time data. A repeated-measures ANOVA revealed significant main effects of group (*F*_(1,16)_ = 7.13, *p* = 0.017, η^2^p = 0.31) and cue (*F*_(2,32)_ = 5.33, *p* = 0.010, η^2^p = 0.25), with no other significant main effects or interactions. Simple effects analyses conducted at the terminal point of training (mean of the last five sessions) showed that sham rats entered the magazine earlier after cue onset on AB+ trials compared with A− (*t*_(8)_ = 2.76, *p* = 0.024, Cohen's *d* = 0.92) and, marginally, B− trials (*t*_(8)_ = 2.22, *p* = 0.058, Cohen's *d* = 0.74). In contrast, latency scores in lesioned rats did not differ significantly across trial types (AB+ vs A−, *t*_(8)_ = 0.07, *p* = 1.00, Cohen's *d* = 0.02; AB+ vs B−, *t*_(8)_ = −0.51, *p* = 1.00, Cohen's *d* = 0.17).

#### PC1

After analyzing all raw response measures, it seems hazardous to conclude that ventral striatal lesions either enhanced or impaired performance on the positive-patterning discrimination. This interpretation aligns with the groups' performance as captured by PC1 scores (Fig. 7*E*). In lesioned rats, PC1 explained on average 69.7% of the variance, compared with 20.3 and 10.0% for PC2 and PC3, respectively. In sham rats, PC1 explained 56.7% of the variance, compared with 27.7 and 15.7% for PC2 and PC3. A repeated-measures ANOVA conducted on PC1 scores revealed a significant main effect of cue (*F*_(2,32)_ = 14.11, *p* < 0.001, η^2^p = 0.47), as well as significant cue × session (*F*_(2,32)_ = 1.60, *p* = 0.019, η^2^p = 0.09) and group × cue interactions (*F*_(2,32)_ = 5.79, *p* = 0.007, η^2^p = 0.27). No other effects were significant. Simple effects analyses focusing on the final five sessions showed that sham rats responded more on AB+ than A− (*t*_(8)_ = 12.5, *p* < 0.002, Cohen's *d* = 4.17) and B− trials (*t*_(8)_ = 3.28, *p* = 0.012, Cohen's *d* = 1.09). In contrast, lesioned rats did not show a reliable discrimination (AB+ vs A−, *t*_(8)_ = 0.99, *p* = 0.354, Cohen's *d* = 0.33; AB+ vs B−, *t*_(8)_ = 0.80, *p* = 0.446, Cohen's *d* = 0.27). Thus, PC1 scores captured the superior discrimination of sham rats, consistent with latency and—most clearly—percent time scores. Importantly, PC1 scores also reflected the weak evidence of discrimination in lesioned rats observed in the number-of-entries measure, which was absent in percent time and latency. The fact that PC1 scores were farther from significance levels compared with number-of-entries scores likely reflects that only three lesioned animals showed evidence of discrimination learning on the latter measure, combined with the relatively small sample size (*n* = 9) for such studies.

Taken together, these findings suggest that when groups differ in how discrimination is expressed across raw measures but not in PC1 scores, the more appropriate conclusion is that the manipulation altered response topography rather than discrimination learning per se. More broadly, they highlight the importance of reporting multiple behavioral measures simultaneously and demonstrate the value of PC1 scores as an accurate composite index of discrimination learning.

## Discussion

The conditioned magazine approach procedure is a cornerstone of behavioral neuroscience. It is the staple procedure for assessing cue–reward learning and is backed by decades of extensive behavioral analysis (Lattal, 1999; Holland, 2000; Bouton and Sunsay, 2001; Gottlieb, 2005; Delamater and Holland, 2008; Harris and Carpenter, 2011; Harris et al., 2013; Harris and Bouton, 2020). However, as we have demonstrated, the utility of the procedure can be compromised by substantial individual differences in response topography. Even under identical training conditions, the behavioral measure that most clearly captures discrimination learning may differ across subjects, and in some cases, animals may even shift their response profile across the course of training. This variability implies that the common practice of reporting a single measure will underestimate the discrimination ability in a subset of animals. Such underestimation has several consequences: It encourages the use of inflated sample sizes, contravening the ethical principle of Reduction in animal research; it increases laboratory costs, which is especially problematic when working with precious animal models or resource-intensive neuroscience methods; and it introduces unnecessary noise into group-level analyses. At the group level, sampling variability may also result in different cohorts of animals expressing discrimination learning in different measures despite receiving identical training, thereby undermining replicability. Finally, reliance on a single measure can mischaracterize the impact of neural manipulations on discrimination learning when such manipulations alter response topography.

To address these issues, we propose monitoring and reporting all standard measures, applying PCA separately within each subject, and using the first principal component (PC1) as a summary index of discrimination learning. Although PC1 generally performs no better than the single behavioral measure that best captures discrimination within a given subject, at the group level, it provides a more equitable summary across animals that express reward expectancy through different response topographies. Conceptually, this approach resembles latent-variable methods such as partial least squares regression (Wold et al., 2001) but differs in that PCA is unsupervised. Thus, in PCA the components are derived solely from the covariance structure of the behavioral measures and are not optimized with respect to cue identity or discrimination performance. As a result, PC1 is constructed not to maximize group differences or cue separation but to capture the dominant axis of variance among the behavioral measures, whether that variance reflects covariance across measures or a strong signal in a single measure. In other words, the method is not designed to cherry-pick results by creating a response variable that maximizes statistical significance on subsequent hypothesis tests.

PCA has proven valuable in related domains involving multiple behavioral measures. It has been used to quantify operant responding (Killeen and Hall, 2001), analyze consummatory behavior in progressive ratio licking tasks (Swanson et al., 2019), examine individual differences in punishment sensitivity (Jean-Richard-Dit-Bressel et al., 2019), and interpret results across learning tasks (Wass et al., 2013) or translational outcome measures (Levin et al., 2013). Nonetheless, it is reasonable to consider whether the present application raises statistical concerns.

Classical inference on population-level principal components (Jolliffe, 2002) assumes independence across observations—an assumption not met when decomposition is performed within subjects across trials. However, our objective is not to infer population-level PCs but to use subject-specific PC scores as summary outcomes in subsequent hypothesis tests. Interpreted in this way, PCA does not violate statistical assumptions, although it does introduce an additional layer of estimation. Because PC1 scores are derived quantities, analyses that treat them as observed outcomes do not explicitly propagate uncertainty associated with estimating subject-specific loadings. Importantly, PCA is a dimension-reduction technique rather than a procedure that mechanically reduces variance. PC1 is defined to capture the maximum variance among linear combinations of the observed measures and therefore does not necessarily exhibit lower variance than any individual raw measure. However, by concentrating variance shared across correlated measures into PC1 and relegating residual variance to higher components, PCA can increase the signal-to-noise ratio of the primary dimension. This reallocation likely contributes to the larger standardized effect sizes observed for PC1 relative to individual raw measures.

While advantageous for sensitivity, failure to account for PC-estimation uncertainty could, in principle, produce mildly anticonservative standard errors, inflated Type I error rates, or confidence intervals with suboptimal coverage. We suspect this risk is limited in the present context, given the large number of trials contributing to each subject-specific PCA and the fact that decomposition is performed independently within subjects rather than at the group level. Nonetheless, the studies presented were not designed as formal calibration tests, and we have not evaluated error control under known null conditions. One possible approach would be to construct confidence intervals using a cluster-bootstrapping procedure (Huang, 2018, and references therein) that resamples at both the subject and trial levels, re-estimates subject-specific PCs, and fits the model to each bootstrap sample. Assessing whether such methods adequately propagate PCA estimation uncertainty would require additional methodological work beyond the scope of the present study. These considerations must be weighed against the limitations of relying on a single behavioral measure.

Additional limitations warrant consideration. First, because PCA is performed separately within each subject, PC1 scores do not represent the same linear combination of measures across animals. This flexibility is advantageous for capturing idiosyncratic response topographies, but it complicates the interpretation of group averages, which summarize subject-specific composites rather than a single fixed metric. Accordingly, the approach does not guarantee that PC1 reflects an identical latent construct or is expressed on a comparable scale in every animal. We therefore emphasize transparent reporting of the underlying raw measures alongside PC1, inspection of subject-specific loading patterns, and verification that PC1 relates monotonically to at least one standard discrimination measure within subjects. Ultimately, the issue of cross-animal construct equivalence parallels an assumption already implicit in the field: that number of entries, percent time, and latency are interchangeable indices of outcome expectancy. If these raw measures are understood to reflect a common construct, then a subject-specific linear combination of them can reasonably be interpreted as indexing that same underlying process.

Second, our suggested approach comes at the cost of reduced interpretability. PCA alters the dependence structure across trials within an animal because each PC1 score is derived from all behavioral measures within that subject. Although this does not inherently violate statistical assumptions in common repeated-measures analyses (e.g., ANOVA, mixed-effects models, and generalized estimating equations), it requires that statistical inferences be expressed in terms of PC1 scores. Undoubtedly, a “20% reduction in PC1 scores after treatment X” is less intuitive than a “20% reduction in the number of head entries.” Once again, to preserve interpretability, it is essential that researchers also monitor and report the direct behavioral measures from which PC1 is computed.

It is also important to consider how PC1 should be interpreted in the absence of discrimination learning. PCA will extract a PC1 for any dataset, including those in which the animal fails to discriminate between rewarded and nonrewarded cues. In such cases, PC1 will simply capture the dominant source of variance in the observed behavioral measures, which need not reflect discrimination-related variance. Crucially, our results suggest that PC1 does not generate spurious cue differences: Animals that failed to discriminate according to all direct measures likewise failed to show discrimination in PC1 scores (Experiment 1). PC1 should therefore be interpreted as a summary of observed behavioral variance, not as an independent indicator of expectancy capable of producing discrimination effects absent from the underlying data.

Third, it should be noted that PCA does not incorporate temporal order. PC1 scores are derived from the covariance structure among measures across observations and do not explicitly model trial-by-trial learning dynamics or within-session state changes. Although PC1 may track learning across sessions, the method is descriptive rather than generative with respect to temporal processes. Researchers primarily interested in dynamic changes in strategy or latent state may benefit from complementary approaches that explicitly model temporal structure, such as state-space models (Smith, 2015, and references therein), hidden Markov models (Rabiner, 1989), or hierarchical latent-variable models (Driver and Voelkle, 2018) with time-varying parameters. Importantly, we have not intended for subject-specific PCA to define a fixed latent expectancy axis that must generalize across independent datasets or future sessions. If covariance structure changes as a result of learning or experimental manipulation, re-estimation of PC loadings is appropriate and consistent with the descriptive objective of the method. Thus, out-of-sample stability is not a prerequisite for its intended use.

While PCA performs well in the present setting, future work could compare it more extensively with alternative statistical methods. For instance, factor models, reduced-rank regression, and partial least squares have been applied in neuroscience to analyze simultaneous recordings across brain regions (Semedo et al., 2020), a problem that also requires joint analysis of multiple related measures. We experimented with other decomposition techniques—including non-negative matrix factorization (Lee and Seung, 2001), archetypal analysis (Cutler and Breiman, 1994), and dynamic factor analysis (Stock and Watson, 2011, 2016)—but these yielded less promising results than PCA in our application. Future studies might also explore longitudinal functional PCA, which can borrow strength across animals while still modeling subject-to-subject variability (Cui et al., 2023). We further tested approaches that incorporate experimental covariates, such as state-space models (Smith, 2015, and references therein), in which hidden states are parameterized as functions of covariates, as well as partial least squares and canonical correlation analysis (Wold et al., 2001; Semedo et al., 2020). State-space models and partial least squares performed reasonably well and warrant further investigation in this context. However, methods of this kind require greater statistical programming expertise and involve modeling decisions that may vary across laboratories, which could limit their broader adoption.

Irrespective of which composite index is used, a key issue remains the source of discordance across raw behavioral measures of magazine approach. Video recordings from our laboratories suggest that magazine entries may reflect diverse behaviors, including checking for reward, head-jerking to auditory cues, or gnawing at the magazine edges (Holland, 1977). Moreover, when checking for reward delivery, rats vary in the depth to which they insert their heads, providing a potential source of discrepancy between number of entries and percent times scores. Rats that fully enter and remain in the magazine on reinforced trials tend to produce percent time scores more sensitive to discrimination learning, whereas rats that hover at the magazine threshold produce number-based scores that better capture their discrimination ability. This suggests that the distance from the snout tip to the back of the magazine might be a particularly sensitive index of approach. Advances in markerless pose estimation, such as DeepLabCut (Nath et al., 2019; Clemensson et al., 2020), now make it feasible to quantify not only the standard approach measures but also additional variables such as distance to the magazine, head orientation, and approach speed—dimensions not detectable by standard sensors (Goedhoop et al., 2023). Incorporating these additional behavioral dimensions may further enhance the value of dimensionality-reduction approaches in generating subject-specific summaries of discrimination learning.

In conclusion, although further validation is warranted, subject-specific PCA proved effective in the present setting while remaining remarkably simple to apply. The method is transparent, requires few modeling choices, and demands little technical expertise. Notably, when applied to just two behavioral measures (e.g., number of entries and percent time), PCA can be implemented without any specialized statistical software or coding. In this case, PC1 scores are mathematically equivalent to the simple average of the two normalized measures on each trial. Although this solution may seem trivial, it consistently performed well in our studies. The equivalence between PCA scores and simple averages in this special case underscores the accessibility of the method. While other statistical procedures may ultimately prove equally or more effective, the relative simplicity of PCA makes it a practical and appealing option for researchers using the conditioned magazine approach procedure.

## Synthesis

Reviewing Editor: Christophe Bernard, INSERM &Institut de Neurosciences des Systèmes Decisions are customarily a result of the Reviewing Editor and the peer reviewers coming together and discussing their recommendations until a consensus is reached. When revisions are invited, a fact-based synthesis statement explaining their decision and outlining what is needed to prepare a revision will be listed below. The following reviewer(s) agreed to reveal their identity: NONE.

Thank you for submitting your manuscript to eNeuro. After careful consideration and review, we have decided to decline publication at this time.

This decision is based on the assessment of a reviewer who was not involved in the previous review process at JN, as well as my own evaluation. The reviewer raised concerns about the novelty and methodological approach of the study, stating: "The authors propose using principal components on multiple dependent variables to reveal differential responding obscured by heterogeneous magazine approach patterns. It is my opinion that this is not an advance as the authors' approach introduces new problems and likely does not offer superior performance over existing approaches."

I share similar concerns. While the manuscript was submitted as a novel tools paper, the primary tool presented is Principal Component Analysis (PCA), a well-established method in both behavioral and neural data analysis. Furthermore, the application of PCA to individual datasets raises methodological issues. Specifically, conducting PCA separately for each dataset can lead to unique solutions that are difficult to compare across animals, potentially hindering the generalizability of the findings.

We understand that this decision may be disappointing. We hope that the feedback provided here, along with the previous reviews from JN, will be helpful as you consider revisions and future submissions.

Full comments from the Reviewer:

In this manuscript, the authors address a potentially widespread issue in the reward learning literature. Appetitive Pavlovian conditioning experiments typically establish a relationship between a cue (or cues) and a food outcome. Behavior directed towards the site of food delivery (magazine) during cue presentation is measured as an index of learning. Additionally, intact individuals are compared to those with some brain manipulation, with roles for brain regions and cell types being ascribed based on observed differences in magazine-directed behavior.

The authors demonstrate that three common magazine measures (rate, percent, and latency) vary within and between individuals. This means that an individual showing a discriminative behavior pattern in one measure can often fail to show discrimination in another, presumably related, measure. This presents a problem. The authors attempt to address this issue by computing all three measures and then performing principal component analysis (PCA) on them. The resulting first component (PC1) describes a large proportion of responding. The authors show that PC1 better captures intact behavior and detects differences in behavior resulting from ventral striatal lesions.

The problem the authors are addressing is significant. However, there are concerns that the principal components approach is neither an advance nor a solution to the problem. These concerns are detailed below.

Concern #1 - One potential solution to the heterogeneous nature of magazine responding is to perform MANOVA on all (three, in this case) dependent measures. This has been done occasionally in the literature but has not been widely adopted. Perhaps the answer is not PCA, but a broader adoption of MANOVA? It is surprising that the authors did not compare the results of ANOVA on PC1 with MANOVA for all the dependent variables. If the results are comparable, it would seem more straightforward to perform MANOVA on the behaviors measured.

Concern #2 - PCA is effective for revealing uncorrelated, low-dimensional features of high-dimensional data. PC1 typically represents the most significant information, which the authors observed in their data. However, an assumption seems to have been made that the magazine behavior reflects a singular learned concept, which can be expressed in various ways. PC1 is then argued to capture this singular learned concept. This assumption may not be valid. Even during simple cue-outcome pairings, rats form many types of associations. Different behaviors may not only be tied to different types of associations but may also be separable results of learning themselves. For example, the authors observed a positive but weak correlation between the percentage of time in the food cup and the number of entries. This might be because these two measures indicate different types of associations being formed or different response strategies. The authors suggest as much in the discussion (head-jerking, rearing, etc.).

Concern #3 - The biggest concern is that relying on PC1 may introduce a whole new set of problems. This is certainly not the authors' goal. Consider the following scenario: The authors perform a brain manipulation (like their ventral striatal lesion) and then assess magazine responding using their three measures. They find that rats with intact brains show excellent discrimination via rate, but not percent or latency. PC1 indicates excellent learning and resembles rate. Rats with brain manipulations show excellent discrimination via latency, but not percent or rate. PC1 indicates excellent learning and resembles latency. ANOVA for PC1 finds no significant main effects or interactions with brain manipulation. Based on the PC1/ANOVA results one would conclude the brain manipulation had no impact on learning/behavior. Would these results be convincing? A MANOVA for the same data would likely return significant group effects or interactions, as it would detect that the measures differed between the two groups. MANOVA would likely find significant group effects or interactions while still observing main effects of cue. Fully embracing PC1 would mean results were blind to such potential differences.

Better solutions to the multiple measures problem are ones already touched on in the manuscript: MANOVA and quantifying behaviors outside of magazine-directed behavior. If interpreting the behavior of two groups is essential, then experimental designs that more strongly "push" animals towards one form of behavioral expression or another should be developed. If behavioral diversity is the goal, then many more behaviors should be quantified.

## Author Response

Response to reviews Editor's concerns:

1) I share similar concerns (to the reviewer). While the manuscript was submitted as a novel tools paper, the primary tool presented is Principal Component Analysis (PCA), a well-established method in both behavioral and neural data analysis.

Response: We agree that PCA is a well-established method in both behavioral and neural data analysis. However, its application to conditioned magazine approach (CMA) data is novel. In the CMA literature, the prevailing practice is to report a single behavioral measure (e.g., number of entries, percent time, or latency), even though these measures often diverge within animals and across groups. Our manuscript introduces the use of subject-specific PC1 scores as a composite index of magazine approach. The novelty lies not in PCA itself, but in demonstrating the risks of relying on a single behavioral measure and in proposing a transparent alternative. We recommend reporting all behavioral measures as well as PC1 scores as a principled summary index of discrimination learning. In doing so, we aim to move the field away from selective reporting toward a method that more faithfully represents each subject's discrimination ability.

2) Furthermore, the application of PCA to individual datasets raises methodological issues. Specifically, conducting PCA separately for each dataset can lead to unique solutions that are difficult to compare across animals, potentially hindering the generalizability of the findings.

Response: We acknowledge that applying PCA separately to each subject produces unique loading patterns that cannot be directly compared across animals. However, this feature is deliberate and central to our approach. Our aim is not to compare loading structures across subjects, but to use each subject's PC1 scores as a summary of the subject's discrimination ability. As we clarify in the manuscript, our hypothesis tests are interpreted on the PC1 scores themselves-not on populationlevel PCs-so generalizability is preserved at the level of the outcome variable.

Importantly, this approach avoids artifacts that could arise if a group-level PCA were performed, since correlation structures among measures vary across individuals. In short, conducting subject-specific PCAs offers a fairer representation of each animal's discrimination ability than selecting any single magazine activity measure, while still allowing valid group-level inference on the resulting PC1 scores.

Full comments from the Reviewer:

In this manuscript, the authors address a potentially widespread issue in the reward learning literature. Appetitive Pavlovian conditioning experiments typically establish a relationship between a cue (or cues) and a food outcome. Behavior directed towards the site of food delivery (magazine) during cue presentation is measured as an index of learning. Additionally, intact individuals are compared to those with some brain manipulation, with roles for brain regions and cell types being ascribed based on observed differences in magazine-directed behavior.

The authors demonstrate that three common magazine measures (rate, percent, and latency) vary within and between individuals. This means that an individual showing a discriminative behavior pattern in one measure can often fail to show discrimination in another, presumably related, measure. This presents a problem. The authors attempt to address this issue by computing all three measures and then performing principal component analysis (PCA) on them. The resulting first component (PC1) describes a large proportion of responding. The authors show that PC1 better captures intact behavior and detects differences in behavior resulting from ventral striatal lesions.

The problem the authors are addressing is significant. However, there are concerns that the principal components approach is neither an advance nor a solution to the problem. These concerns are detailed below.

Concern #1 - One potential solution to the heterogeneous nature of magazine responding is to perform MANOVA on all (three, in this case) dependent measures. This has been done occasionally in the literature but has not been widely adopted. Perhaps the answer is not PCA, but a broader adoption of MANOVA? It is surprising that the authors did not compare the results of ANOVA on PC1 with MANOVA for all the dependent variables. If the results are comparable, it would seem more straightforward to perform MANOVA on the behaviors measured.

Response: We appreciate the reviewer's suggestion regarding MANOVA. We now explain in the Introduction why MANOVA is rarely a viable option in conditioned magazine approach studies. For convenience, the relevant passage is included below:

When multiple quantitative dependent variables are measured on observations nested within groups, a common approach in the behavioral sciences is multivariate analysis of variance (MANOVA). Compared with running separate ANOVAs, MANOVA can offer greater power and can detect associations among dependent variables. Like multiple regression and discriminant analysis, however, MANOVA is sensitive to multicollinearity, and therefore its use is limited in situations where the number of variables approaches or exceeds the number of observations. In repeated-measures studies with many within-subject conditions-such as factorial combinations of cues and sessions, as is typical in conditioned magazine-approach studies-wide-format datasets create a separate column for each condition, rapidly inflating the number of variables relative to subjects. This inflation increases the risk of multicollinearity, which precludes the analysis, or of near multicollinearity, which provides unstable parameters estimates.

To further complicate matters, MANOVA requires nonzero variance within each group for every dependent variable. If all animals respond similarly to a particular cue on a given measure (e.g., by not responding to a nonreinforced cue), this measure will have zero variance and effectively become a constant, causing the MANOVA to fail. Even when this is not the case, conditioned magazine approach data often violate additional MANOVA assumptions: 1) the behavioral measures are typically correlated-a pattern leading to multicollinearity; 2) the measures rarely follow a multivariate normal distribution because they include bounded (e.g., percent time) or skewed variables; and 3) the assumption of homogeneous covariance matrices across groups is unlikely to hold when neural manipulations change the variability and interdependence of measures. Together, these issues make MANOVA impractical for many conditioned magazine approach designs- which may explain why it is rarely used in the literature (for an exception, see McDannald et al., 2011).

Concern #2 - PCA is effective for revealing uncorrelated, low-dimensional features of highdimensional data. PC1 typically represents the most significant information, which the authors observed in their data. However, an assumption seems to have been made that the magazine behavior reflects a singular learned concept, which can be expressed in various ways. PC1 is then argued to capture this singular learned concept. This assumption may not be valid. Even during simple cue-outcome pairings, rats form many types of associations. Different behaviors may not only be tied to different types of associations but may also be separable results of learning themselves. For example, the authors observed a positive but weak correlation between the percentage of time in the food cup and the number of entries. This might be because these two measures indicate different types of associations being formed or different response strategies. The authors suggest as much in the discussion (head-jerking, rearing, etc.).

Response: We agree that PCA is generally used to extract low-dimensional features from high-dimensional data, and we acknowledge the reviewer's important point that magazine responding may reflect multiple associative processes. However, the prevailing assumption in the conditioned magazine approach literature has long been that all three measures-entries, percent time, and latency-index the same latent construct: reward expectancy. This assumption underlies the widespread practice of reporting only a single measure, often whichever best demonstrates discrimination in a given dataset. Our contribution is to highlight the pitfalls associated with this practice and to propose a more principled and transparent alternative.

In our experience, the moderate correlations among measures largely reflect differences in response style. For example, rats that insert their heads deeply into the magazine tend to accrue high percent-time scores but relatively few entries, whereas rats that hover at the threshold generate more entries but less sustained occupancy. The reviewer raises the intriguing possibility that such differences could also reflect distinct associative processes involving the cue and various aspects (e.g., sensory, affective, or motor) of the outcome representation. We agree that this is an important avenue for future work. Importantly, however, our method does not assume that all response profiles stem from a single type of association, nor do we propose replacing the raw measures with PC1 scores alone. Rather, we advocate reporting all measures alongside PC1 scores, and using the latter only as a subject-specific summary of discrimination learning. If heterogeneous associative processes do indeed contribute to different response profiles, our approach will make such differences more detectable than the current practice of reporting a single measure.

Concern #3 - The biggest concern is that relying on PC1 may introduce a whole new set of problems. This is certainly not the authors' goal. Consider the following scenario: The authors perform a brain manipulation (like their ventral striatal lesion) and then assess magazine responding using their three measures. They find that rats with intact brains show excellent discrimination via rate, but not percent or latency. PC1 indicates excellent learning and resembles rate. Rats with brain manipulations show excellent discrimination via latency, but not percent or rate. PC1 indicates excellent learning and resembles latency.

ANOVA for PC1 finds no significant main effects or interactions with brain manipulation.

Based on the PC1/ANOVA results one would conclude the brain manipulation had no impact on learning/behavior. Would these results be convincing? A MANOVA for the same data would likely return significant group effects or interactions, as it would detect that the measures differed between the two groups. MANOVA would likely find significant group effects or interactions while still observing main effects of cue. Fully embracing PC1 would mean results were blind to such potential differences.

Better solutions to the multiple measures problem are ones already touched on in the manuscript: MANOVA and quantifying behaviors outside of magazine-directed behavior. If interpreting the behavior of two groups is essential, then experimental designs that more strongly "push" animals towards one form of behavioral expression or another should be developed. If behavioral diversity is the goal, then many more behaviors should be quantified.

Response: In our response to Concern #1, we have already explained why MANOVA is often not a viable option in this context. However, we appreciate the reviewer's concern. To clarify, we are not proposing that PC1 scores replace the traditional direct measures of magazine approach. Rather, our recommendation is that researchers report all direct measures and use subject-specific PC1 scores as a complementary summary index. This distinction is now further clarified in the manuscript (see pasted section below).

Importantly, the scenario raised by the reviewer is precisely the type of situation our approach is designed to address. If two groups differ in how discrimination is expressed across raw measures, relying on a single measure could lead to misleading conclusions about learning ability. By reporting both the raw measures and PC1 scores, researchers can distinguish between changes in response topography and true changes in discrimination learning. This is now more explicitly state in the MS:

Taken together, these findings suggest that when groups differ in how discrimination is expressed across raw measures but not in PC1 scores, the more appropriate conclusion is that the manipulation altered response topography rather than discrimination learning per se. More broadly, they highlight the importance of reporting multiple behavioral measures simultaneously and demonstrate the value of PC1 scores as an accurate composite index of discrimination learning.

On the reviewer's final point : "If interpreting the behavior of two groups is essential, then experimental designs that more strongly 'push' animals toward one form of behavioral expression or another should be developed. If behavioral diversity is the goal, then many more behaviors should be quantified." Response: We would like to clarify that, at present, there is no known way to design experiments that reliably "push" subjects toward a particular response topography. As our data show, considerable variability in the expression of discrimination learning emerges even under identical training conditions. Nonetheless, by reporting multiple direct measures as we recommend, researchers will be better positioned to identify the training parameters that systematically influence response topography and, ultimately, to design their experiments accordingly.
